# Incident Instrumental Activities of Daily Living Difficulty in Older Adults: Which Comes First? Findings From the Advanced Cognitive Training for Independent and Vital Elderly Study

**DOI:** 10.3389/fneur.2020.550577

**Published:** 2020-10-22

**Authors:** Danielle M. Feger, Sherry L. Willis, Kelsey R. Thomas, Michael Marsiske, George W. Rebok, Cynthia Felix, Alden L. Gross

**Affiliations:** ^1^Center on Aging and Health, Johns Hopkins University Bloomberg School of Public Health, Baltimore, MD, United States; ^2^Department of Epidemiology, Johns Hopkins University Bloomberg School of Public Health, Baltimore, MD, United States; ^3^Department of Psychiatry and Behavioral Sciences, University of Washington School of Medicine, Seattle, WA, United States; ^4^Veterans Affairs San Diego Healthcare System, San Diego, CA, United States; ^5^Department of Psychiatry, University of California San Diego School of Health Sciences, La Jolla, CA, United States; ^6^Department of Clinical and Health Psychology, University of Florida College of Public Health and Health Professions, Gainesville, FL, United States; ^7^Department of Mental Health, Johns Hopkins University Bloomberg School of Public Health, Baltimore, MD, United States; ^8^Department of Epidemiology, University of Pittsburgh Graduate School of Public Health, Pittsburgh, PA, United States

**Keywords:** IADLs, older adults, activites of daily living, MEPSUM, cognitive training

## Abstract

**Introduction:** Instrumental activities of daily living (IADLs) are complex daily tasks important for independent living. Many older adults experience difficulty with IADLs as their physical and/or cognitive function begins to decline. However, it is unknown in what order IADLs become difficult.

**Methods:** Participants from the Advanced Cognitive Training for Independent and Vital Elderly (ACTIVE) study who were free of IADL difficulty at baseline (*N* = 1,277) were followed up to 10 years until first reported IADL difficulty. A total of 19 IADL tasks were grouped into seven task categories. A discrete-time multiple-event process survival mixture model (MEPSUM) was used to generate hazard estimates of incident IADL difficulty in seven groups from ages 65 to 80. Hazard estimates were compared in the three intervention groups (memory, inductive reasoning, and speed of information processing) vs. the no-contact control group.

**Results:** A total of 887 (69.5%) participants reported incident difficulty in at least one IADL task category. Compared to individuals who remained free of IADL difficulty, those who reported incident difficulty were more likely to be older, female, and have lower Short Form 36 general health scores. The IADL task categories to first become difficult were housework, managing health care, and phone use. There were no differences by intervention group in the hazard estimates of incident IADL difficulty.

**Conclusion:** Managing health care and phone use are more cognitively demanding IADLs, and individuals who experience difficulty in these tasks first may be more likely to experience cognitive decline. Recognizing early difficulty in managing health care may allow for implementation of compensation strategies to minimize unintentional medication misuse, increased adverse medical events, and unnecessary hospitalization. Training of a specific cognitive domain may not influence ordering of IADL difficulty because IADL tasks require proficiency in, and integration of, multiple cognitive domains.

## Introduction

Difficulty in performing daily activities increases with age (1, 2) due to declining physical and cognitive functioning. Maintaining functional independence is of great importance for older adults (3) and is associated with increased quality of life (4) and lower health care expenditures (5). Common everyday activities are categorized into two groups: instrumental activities of daily living (IADLs) and basic activities of daily living (ADLs). IADLs encompass more complex tasks important for independent living (e.g., cooking, household chores, and handling money) (6), while ADLs are basic with more physical tasks of self-care necessary for independent living, and include functions such as bathing, dressing, and feeding (7). Because IADL tasks are more complex, most older adults experience difficulty with some IADLs before they experience difficulty with ADLs (8).

Prior research has evaluated the hierarchical progression of ADL difficulty. Katz et al. (7), who developed the original ADL scale, theorized that loss of ADL function mirrored the developmental achievement of ADLs in young children: skills that are obtained first in childhood such as self-feeding are lost last in older adults. Subsequent studies have generally found patterns consistent with this hypothesis, with mobility and bathing usually the first ADLs to become difficult, followed by transferring, dressing, toileting, and feeding becoming difficult last (9–11). However, among community-dwelling older adults, patterns of progressive difficulty are varied (12, 13).

In contrast to ADL difficulty, the relative order in which IADLs become difficult has not been well-studied. Studies of progression of IADL difficulty often consider only a limited set of IADLs (14, 15), increasing counts of difficult IADLs (16–18), or using one scale that fails to distinguish IADLs from ADLs (19–22). These techniques do not account for relationships among IADLs and may miss transitional patterns (23). Because IADL performance is thought to reflect underlying cognitive and physical function (24, 25), identifying early incident IADL difficulty may facilitate earlier intervention to maintain remaining function.

The primary objective of this study was to determine the relative ordering of incident difficulty across 19 IADLs representing seven task groups (preparing meals, housework, managing finances, managing health care, phone use, shopping, and travel outside of the home) in a large sample of high-functioning, community-living older adults. The secondary objective was to determine whether this relative ordering differed in individuals receiving cognitive training relative to a control group. We hypothesize that older adults, on average, first report problems with more cognitively demanding IADLs including managing finances, managing medications, and managing health care.

While previous studies within the ACTIVE cohort have shown that cognitive intervention improved ability on performance-based IADL measures (26, 27), we did not expect to observe any differences in first incident IADL difficulty in individuals receiving cognitive training compared to those not receiving cognitive training.

## Materials and Methods

### Study Sample

We examined data from the Advanced Cognitive Training for Independent and Vital Elderly (ACTIVE) study. Methods of the ACTIVE study have been described elsewhere (27). Briefly, *N* = 2,802 community-living older adults aged 65 years and older were recruited from six US geographical sites beginning in 1998, randomized to a cognitive training intervention, and followed for up to 10 years with in-person visits. Participants were randomized to receive one of three cognitive interventions (memory, inductive reasoning, speed of information processing) or a no-contact control group. The primary outcome of interest in the original ACTIVE study was everyday functioning, as defined by IADLs. Secondary outcomes of interest for the original ACTIVE study included everyday processing speed and driving habits. Data collection occurred at baseline, immediately post-training at 10 weeks, and at follow-up at years 1, 2, 3, 5, and 10. For this analysis, the primary outcome was first incident IADL difficulty; only participants free of all IADL difficulty items at baseline were included (*N* = 1,277; 45.6% of total sample) without regard to their ADL status. Because IADLs were not assessed at the immediate post-training visit, this visit was excluded from the present study. Each study site's local institutional review board approved the ACTIVE study.

### Variables

Participants' self-reported IADL ability for 19 tasks at each study visit (**Table 2**). For each task, participants were asked, (1) “In the last 7 days, how much of the activity did you do on your own?” and (2) “How difficult was it (or would it have been) to do on your own?” Possible responses for (1) included 1—Did all on own; 2—Some help some of the time; 3—Help all of the time; 4—Fully performed by others; and 5—Activity not performed by you or others. Possible responses for (2) included 1—Not difficult; 2—Some help needed or I am slow, or I became tired; 3—Great difficulty. Participants' response to (2) was the primary outcome of this analysis, and a response of 2 or 3 constituted having difficulty with the specific task.

### Adjustment Variables

Both unadjusted and adjusted analyses were performed. Adjustment variables included age at study entry, sex, race (white, black, or other), years of education, and baseline self-reported global health. The global health score was calculated using the MOS Short Form 36 (28) (SF-36) general health subscale and ranges from 0 (worst) to 100 (best).

### Analysis Plan

Means and proportions were used to describe baseline demographic and health characteristics, and *t*-tests and chi-square tests were used to evaluate for differences between those who ever reported any incident IADL difficulty during the study and those who never did. Participants were followed until first incident difficulty with any IADL; more than one IADL could become newly difficult at the same study visit. The proportion of participants reporting incident IADL difficulty at each follow-up visit was also calculated.

The 19 IADLs assessed in ACTIVE were *a priori* grouped into seven categories of related tasks for model estimation and ease of interpretation of findings (**Table 2**). Patterns of relative worsening in IADL groups were evaluated using a discrete-time multiple-event process survival mixture (MEPSUM) model (29). To use a biologically relevant timescale, we aligned participant follow-up by chronological age instead of study follow-up time and incorporated left-hand censoring for individuals (30). Advantages of the MEPSUM model over traditional proportional hazards survival models include accommodation of multiple non-repeated events that may be reported simultaneously during discrete study visits, thereby accounting for ties between different categories of IADLs. Results from the MEPSUM model are provided as the probability of incident IADL difficulty for each task at each year of age, which may be interpreted equivalently to hazard estimates derived using standard univariate discrete-time survival analysis methods (29, 31). All models were estimated using ages 66–80 years to aid in model fitting.

To statistically test which IADLs were more likely to occur first, McNemar's test (32) was applied to each possible pairing of IADL task, for each age from 66 to 80 years. For each pairing, the number of participants who experienced difficulty in one task, both tasks, or none was calculated. Discordant pairs (difficulty in one task only) represent individuals for whom only one IADL was newly difficult (and thus became difficult first). For each pairing, a task in which a significantly larger percentage of participants reported difficulty in only that task indicates that a specific task was more likely to become difficult first relative to the other tasks.

A second MEPSUM model was constructed to evaluate differences in the probability of incident IADL difficulty comparing the intervention groups (memory, inductive reasoning, and speed of processing) separately relative to the control group. Because the absolute number of incident IADL difficulties within each intervention group was small, age was grouped into intervals of 5 years to facilitate model fitting (ages 66–70, 71–75, and 76–80). Odds ratios and 95% confidence intervals comparing the probability (hazard) estimates between each of the three intervention groups relative to the control group were generated automatically using the cinterval command in Mplus.

Descriptive analyses and McNemar's tests were performed using Stata version 15.1 (StataCorp 2017, College Station, TX, USA). MEPSUM analyses were estimated in Mplus version 8.2 (Muthén & Muthén 2018, Los Angeles, CA, USA) using a maximum likelihood estimator with 300 initial stage random starts and 60 final stage optimizations. Results with *p* < 0.05 were considered statistically significant.

## Results

### Descriptive Statistics

Of the *N* = 1,277 participants included who had no prevalent IADL difficulty, most were white (73.9%), female (77.0%), and the average age at baseline was 72.8 ± 5.5 years. The majority of participants were either married (36.0%) or widowed (40.6%). The average years of education completed was 13.67 ± 2.63 years, and the average SF-36 general health score at baseline was 72.27 ± 18.20. The median time to a first incident IADL difficulty was 5 years. Compared to ACTIVE participants who entered the study reporting difficulty with at least one IADL (*N* = 1,525, 54.4%), participants free of IADL difficulty at baseline were, on average, younger (72.8 vs. 74.3 years; *p* < 0.001), had higher education (13.67 vs. 13.41 years; *p* = 0.014), higher SF-36 general health scores (74.07 vs. 64.66; *p* < 0.001), and were more likely to be white (71.1 vs. 54.8%; *p* = 0.032). No difference in marital status (*p* = 0.314) or sex (77.0 vs. 75.0% female; *p* = 0.212) between groups was observed.

**Figure 1:**
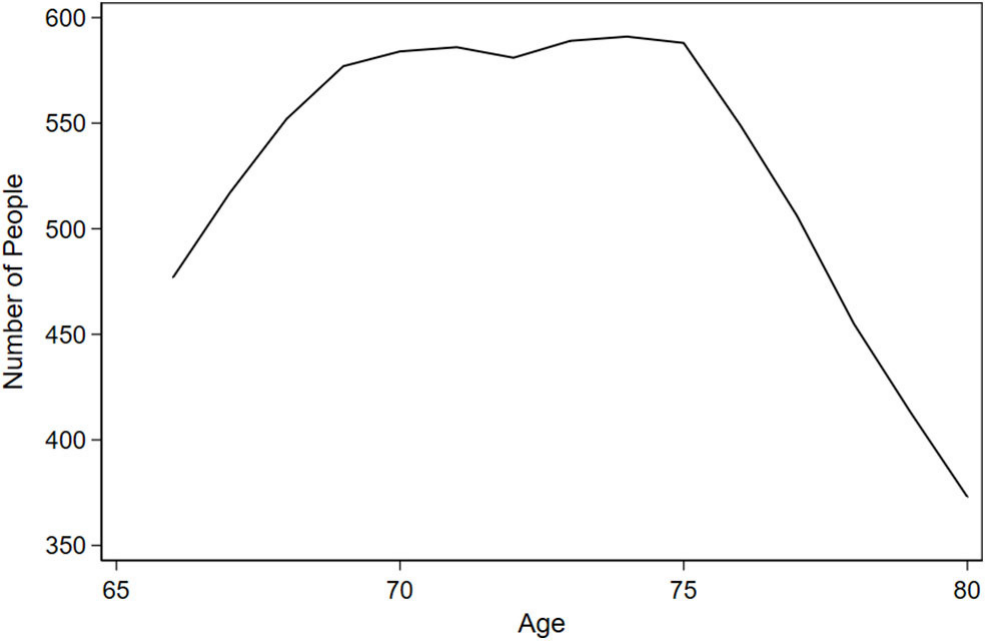
Number of people contributing data at each year of age: results from Advanced Cognitive Training for Independent and Vital Elderly (ACTIVE) (*N* = 1,277).

Of the *N* = 1,277 participants free of IADL difficulty at baseline, 887 (69.5%) developed incident difficulty with any IADL task over 10 years of follow-up. Figure 1 shows the distribution of people contributing data by age. For most (59.6%), the first incident IADL difficulty occurred alone, and for another 19.1% the first incident difficulty occurred with two tasks at the same visit. For the remainder of participants (21.3%), the first incident difficulty occurred with more than two tasks at the same visit. Compared to participants who remained free of incident IADL difficulty throughout the study, participants with incident IADL difficulty were older (73.52 vs. 71.18 years; *p* < 0.001), more likely to be female (78.8 vs. 72.8%; *p* = 0.019), had worse SF-36 global health scores at baseline (69.92 vs. 77.75; *p* < 0.001), and had lower total years of education (13.54 vs. 13.96; *p* = 0.007). Those who experienced incident IADL difficulty were also more likely to be widowed at baseline (43.4 vs. 34.1%; *p* = 0.010) (Table 1).

**Table 1:** Demographic characteristics of the Advanced Cognitive Training for Independent and Vital Elderly (ACTIVE) sample free of instrumental activities of daily living (IADL) difficulty at baseline.

**Variable *N* (%) or Mean (SD)**	**Overall (*N* = 1,277)**	**Experienced incident IADL difficulty (*N* = 887)**	**Did not experience incident IADL difficulty (*N* = 390)**	***P*-value for difference**
Intervention assignment				0.918
Memory	329 (25.8%)	228 (25.7%)	101 (25.9%)	
Reasoning	306 (24.0%)	214 (24.1%)	92 (23.6%)	
Speed	326 (25.5%)	222 (25.0%)	104 (26.7%)	
Control	316 (24.7%)	223 (25.1%)	93 (23.8%)	
Age at baseline, years	72.81 (5.48)	73.52 (5.63)	71.18 (4.73)	<0.001
Female	983 (77.0%)	699 (78.8%)	284 (72.8%)	0.019
Race				0.244
White	943 (73.8%)	667 (75.2%)	276 (70.8%)	
Black	321 (25.1%)	211 (23.8%)	110 (28.2%)	
Other	13 (1.0%)	9 (1.0%)	4 (1.0%)	
Years of education	13.67 (2.63)	13.54 (2.66)	13.96 (13.71)	0.007
BMI	28.44 (5.37)	28.55 (5.53)	28.19 (4.97)	0.270
Diabetes	152 (11.9%)	110 (12.4%)	42 (10.8%)	0.399
Hypertension	622 (48.9%)	443 (50.1%)	179 (46.0%)	0.178
Baseline SF-36	72.27 (18.20)	69.92 (18.27)	77.75 (16.82)	<0.001
Marital status				0.01
Married	460 (36.0%)	311 (35.1%)	149 (38.2%)	
Separated	15 (1.2%)	9 (1.0%)	6 (1.5%)	
Divorced	194 (15.2%)	130 (14.7%)	64 (16.4%)	
Widowed	518 (40.6%)	385 (43.4%)	133 (34.1%)	
Single	89 (7.0%)	52 (5.9%)	37 (9.5%)	

*SD, standard deviation; IADL, Instrumental Activities of Daily Living*.

*P-values indicate the result of the statistical test of differences between individuals who experienced incident IADL difficulty and individuals who did not experience incident IADL difficulty*.

Table 2 shows the number of incident difficult IADLs by study visit. Although most participants developed incident difficulty at some point during the study, no one task accounted for the majority of incident cases. The first task to become difficult was quite heterogenous in this population. The most common IADLs to become difficult first were giving self-injections, applying ointments, and changing bandages, representing 13.76% of all new incident cases. The second most common IADL to become difficult first was doing dishes, dusting, making beds, and tidying up (13.37%), and the third most common IADL to become difficult was remembering often-called numbers without having to look them up (11.05%). The IADL task least likely to become difficult first was hanging up at the end of a phone call (0.77%), followed by answering the phone (1.22%), and keeping household expenses balanced (2.93%).

**Table 2:** Number (%) of incident difficult IADLS, by study visit: descriptive results from ACTIVE (*N* = 1,277).

**IADL task**	**Year 1 *N* (%)**	**Year 2 *N* (%)**	**Year 3 *N* (%)**	**Year 5 *N* (%)**	**Year 10 *N* (%)**
**Task group 1: preparing meals**					
Planning meals, reading recipes, assembling ingredients	31 (2.68%)	18 (2.08%)	6 (0.89%)	11 (1.92%)	43 (10.94%)
Setting out food and utensils	21 (1.81%)	10 (1.16%)	4 (0.60%)	7 (1.22%)	29 (7.38%)
Cooking	33 (2.85%)	14 (1.62%)	7 (1.04%)	11 (1.92%)	38 (9.67%)
**Task group 2: housework**					
Doing dishes, dusting, making beds, tidying up	82 (7.08%)	44 (5.09%)	32 (4.77%)	29 (5.05%)	55 (13.99%)
Laundry	48 (4.15%)	20 (2.31%)	12 (1.79%)	18 (3.14%)	40 (10.18%)
**Task group 3: managing finances**					
Handling money, writing checks	12 (1.04%)	5 (0.58%)	5 (0.75%)	7 (1.22%)	25 (6.36%)
Ensuring that all bills are paid on time	12 (1.04%)	7 (0.81%)	7 (1.04%)	9 (1.57%)	27 (6.87%)
Balancing checkbooks	42 (3.63%)	27 (3.13%)	14 (2.09%)	17 (2.96%)	34 (8.65%)
Keeping household expenses balanced	8 (0.69%)	6 (0.69%)	4 (0.60%)	7 (1.22%)	28 (7.12%)
**Task group 4: managing health care**					
Keeping track of doctor appointments	11 (0.95%)	6 (0.69%)	6 (0.89%)	6 (1.05%)	27 (6.87%)
Remembering to take medications on time as prescribed by a doctor	27 (2.33%)	10 (1.16%)	8 (1.19%)	10 (1.74%)	30 (7.63%)
Opening medicine bottles, taking own medications	37 (3.20%)	13 (1.50%)	14 (2.09%)	22 (3.83%)	45 (11.45%)
Giving self-injections, applying ointments, changing bandages	77 (6.65%)	46 (5.32%)	31 (4.62%)	33 (5.75%)	62 (15.78%)
**Task group 5: phone use**					
Looking up phone numbers—either by phone books or by calling “information”	21 (1.81%)	11 (1.27%)	9 (1.34%)	10 (1.74%)	24 (6.11%)
Remembering often called numbers without having to look them up	64 (5.53%)	38 (4.40%)	31 (4.62%)	22 (3.83%)	45 (11.45%)
Answering phone when someone calls	5 (0.43%)	3 (0.35%)	2 (0.30%)	1 (0.17%)	11 (2.80%)
Hanging up at end of call	1 (0.09%)	1 (0.12%)	0 (0.00%)	1 (0.17%)	11 (2.80%)
**Task group 6: shopping**					
Shopping for food and household items	42 (3.63%)	15 (1.74%)	16 (2.38%)	17 (2.96%)	57 (14.50%)
**Task group 7: travel**					
Travel by vehicle to go to places beyond walking distances	24 (2.07%)	13 (1.50%)	11 (1.64%)	12 (2.09%)	54 (13.74%)
Total N at Risk	1158	864	671	574	393

*IADL, Instrumental Activities of Daily Living*.

### Main Findings

**Figure 2:**
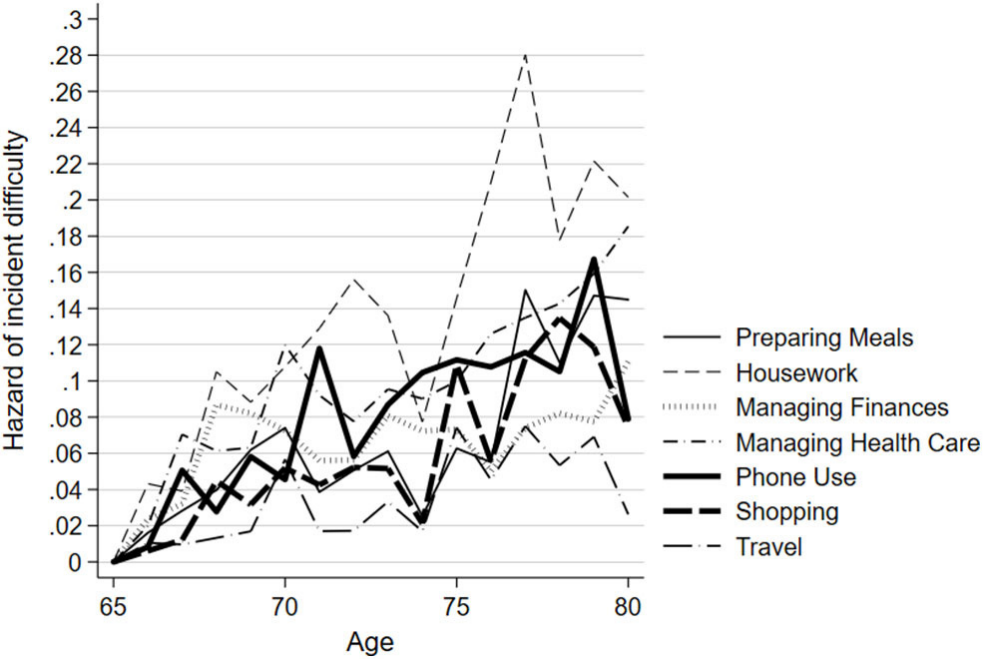
Hazard of incident IADL difficulty by task group, adjusted model: results from ACTIVE (*N* = 1,277). Results of discrete-time multiple-event process survival mixture model (MEPSUM) model, as described in the Methods section. Figure shows the hazard estimates of incident difficulty in seven Instrumental Activities of Daily Living (IADL) task groups as a function of age. The hazards are estimated from a model with age 65 years as the time origin.

**Figure 3:**
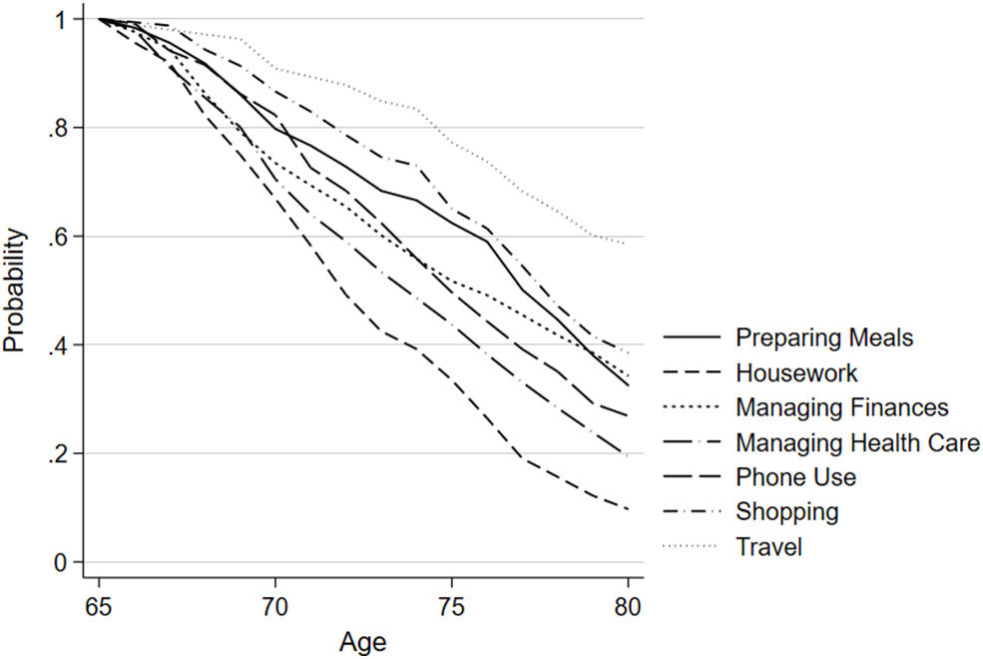
Probability of remaining free of IADL difficulty by task group, adjusted model: results from ACTIVE (*N* = 1,277). Results of MEPSUM model, as described in the Methods section. Figure shows the probability of remaining free of difficulty in seven Instrumental Activities of Daily Living (IADL) task groups as a function of age. This survival probability is derived using model hazard estimates in the standard discrete time survival formula [see Ref. (28)].

Figures 2, 3 show the estimated probability of incident IADL difficulty at each year of age, for each IADL task group from the adjusted model. Both unadjusted and adjusted MEPSUM models containing one latent class were estimated. For the unadjusted model, the number of free parameters was 103, the log likelihood (LL) was −4,611.281, the AIC was 9,428.562, and the BIC was 9,949.082. For the adjusted model, the number of free parameters was 138, the LL was −4,384.630, the AIC was 9,045.260, and the BIC was 9,737.923. The estimated probability may be interpreted as the hazard of incident IADL difficulty, which is the probability of an individual experiencing difficulty in a specific IADL at a specific age given that they have remained free of any IADL difficulty up until that age. Findings of the adjusted model are as follows: Although the majority of participants experienced incident difficulty at some point during the study, the probability of experiencing new difficulty at any 1 year of age was low due to the wide dispersion of age at baseline. The hazard of experiencing difficulty with managing finances and traveling was both low and constant across all age groups. Difficulty with preparing meals and shopping increased beginning around the age of 75. Most notable is the steady increase in the probability of incident difficulty with housework, managing health care, and phone use with age. In the unadjusted model, managing health care was the IADL with the highest hazard of becoming difficult first, followed by housework, and phone use (see Supplemental Material). In the adjusted model, housework was the IADL with the highest hazard of becoming difficult first, followed by managing health care and phone use.

Results from McNemar's test to statistically test which tasks occurred earlier are in Table 3. Difficulty preparing meals was significantly less likely to occur before difficulty performing housework [ratio range (0.19–0.67)], managing health care [ratio range (0.11–0.46)], or using the phone [ratio range (0.12–1.000)]. Difficulty performing housework was significantly more likely to occur before difficulty managing money [ratio range (0.88–3.86)], shopping [ratio range (1.27–4.20)], and traveling [ratio range (1.43–8.00)], but less likely to become difficult before managing health care [ratio range (0.37–1.13)]. Difficulty managing money was less likely to occur before managing health care [ratio range (0.25–0.82)] or using the phone [ratio range (0.30–2.25)], while managing health care was more likely to become difficult before using the phone [ratio range (0.90–3.57)], shopping [ratio range (1.91–12.00)], or traveling [ratio range (3.00–13.50)]. Finally, phone use was more likely to become difficult before shopping [ratio range (1.00–8.50)] and traveling [ratio range (1.00–10.00)].

**Table 3:** Odds ratio of each IADL becoming difficult first: results from McNemar's test.

**Age**	**Preparing meals**						**Housework**					**Money**			
	**Housework**	**Money**	**Health**	**Phone**	**Shop**	**Travel**	**Money**	**Health**	**Phone**	**Shop**	**Travel**	**Health**	**Phone**	**Shop**	**Travel**
66	0.33	0.5	0.33	1	.	1	1.5	1	3	.	3	0.67	2	.	2
67	0.67	0.67	**0.17**	0.29	2	2	1	0.25	0.43	3	3	**0.25**	0.43	3	3
68	0.33	0.33	0.27	0.75	0.75	.	1	0.82	2.25	2.25	.	0.82	2.25	2.25	.
69	0.56	0.56	**0.36**	0.56	1.67	2.5	1	0.64	1	3	**4.5**	0.64	1	3	4.5
70	0.6	0.75	**0.24**	0.86	1.2	0.86	1.25	**0.4**	1.43	2	1.43	**0.32**	1.14	1.6	1.14
71	**0.23**	0.5	**0.17**	**0.15**	0.75	1.5	2.17	0.72	0.65	**3.25**	**6.5**	**0.33**	**0.3**	1.5	3
72	**0.31**	0.83	**0.29**	0.42	0.83	2.5	**2.67**	0.94	1.33	**2.67**	**8**	**0.35**	0.5	1	3
73	0.38	0.56	**0.26**	**0.33**	1	1.25	1.44	0.68	0.87	**2.6**	**3.25**	0.47	0.6	1.8	2.25
74	0.29	0.25	**0.11**	**0.12**	1	1	0.88	**0.37**	0.41	3.5	3.5	**0.42**	0.47	4	4
75	**0.36**	0.63	**0.24**	**0.28**	0.45	0.56	1.75	0.67	0.78	1.27	1.56	**0.38**	0.44	0.73	0.89
76	**0.19**	0.67	**0.17**	**0.25**	0.8	0.8	**3.5**	0.88	1.31	**4.2**	**4.2**	**0.25**	**0.38**	1.2	1.2
77	**0.41**	1.57	**0.46**	0.69	1.1	1.38	**3.86**	1.13	1.69	**2.7**	**3.38**	**0.29**	**0.44**	0.7	0.88
78	**0.5**	1	**0.29**	0.54	0.64	1.4	**2**	0.58	1.08	1.27	**2.8**	**0.29**	0.54	0.64	1.4
79	0.53	1.5	**0.38**	**0.45**	1	1.5	**2.83**	0.71	0.85	1.89	**2.83**	**0.25**	**0.3**	0.67	1
80	0.57	1	**0.3**	1	1.6	4	1.75	**0.52**	1.75	**2.8**	**7**	**0.3**	1	1.6	4
**Age**	**Health**						**Phone**					**Shop**			
	**Phone**	**Shop**	**Travel**				**Shop**	**Travel**				**Travel**			
66	3	.	3				.	1				0			
67	1.71	**12**	**12**				7	7				1			
68	2.75	2.75	.				1	.				.			
69	1.56	**4.67**	**7**				3	4.5				1.5			
70	**3.57**	**5**	**3.57**				1.4	1				0.71			
71	0.9	**4.5**	**9**				**5**	**10**				2			
72	1.42	**2.83**	**8.5**				2	**6**				3			
73	1.27	**3.8**	**4.75**				**3**	**3.75**				1.25			
74	1.12	**9.5**	**9.5**				**8.5**	**8.5**				1			
75	1.17	1.91	**2.33**				1.64	2				1.22			
76	1.5	**4.8**	**4.8**				**3.2**	**3.2**				1			
77	1.5	**2.4**	**3**				1.6	2				1.25			
78	1.85	**2.18**	**4.8**				1.18	**2.6**				2.2			
79	1.2	**2.67**	**4**				**2.22**	**3.33**				1.5			
80	**3.38**	**5.4**	**13.5**				1.6	4				2.5			

*IADL, Instrumental Activities of Daily Living*.

*The odds ratio (OR) of the header IADL becoming difficult first relative to secondary (vertically oriented) IADL is displayed; statistically significant results are displayed in bold. OR <1 indicates that the header task was less likely to become difficult first compared to the secondary task. OR > 1 indicates that the header task was more likely to become difficult first compared to the secondary task*.

### Effect of Cognitive Intervention

In the secondary analysis, there were no statistically significant differences in the hazard probability in any of the intervention groups compared to the control group, with the exception that individuals in the inductive reasoning group were less likely to experience incident difficulty with travel between ages 71–75 years relative to those in the control group [OR = 0.47 (0.16–1.39), *p* = 0.039] (Table 4).

**Table 4:** Comparison of each ACTIVE intervention group to control group.

**IADL task group**	**Ages 66–70**		**Ages 71–75**		**Ages 76–80**	
**Memory vs. Control**	**OR**	**(95% CI)**	**OR**	**(95% CI)**	**OR**	**(95% CI)**
Preparing meals	1.29	(0.30, 5.55)	1.75	(0.68, 4.56)	1.61	(0.78, 3.32)
Housework	1.36	(0.80, 2.30)	2.92	(0.93, 9.23)	2.08	(1.13, 3.84)
Managing finances	1.00	(0.67, 1.49)	1.06	(0.71, 1.56)	0.87	(0.30, 2.49)
Managing health care	0.86	(0.51, 1.45)	0.82	(0.50, 1.35)	0.87	(0.53, 1.41)
Phone use	0.91	(0.61, 1.36)	0.75	(0.52, 1.10)	0.76	(0.52, 1.09)
Shopping	1.20	(0.65, 2.21)	1.01	(0.63, 1.62)	0.81	(0.53, 1.24)
Travel	3.96	(0.45, 34.57)	1.65	(0.73, 3.73)	1.37	(0.73, 2.57)
**Reasoning vs. Control**						
Preparing meals	1.24	(0.27, 5.72)	1.39	(0.52, 3.73)	2.05	(1.01, 4.13)
Housework	1.42	(0.83, 2.41)	0.92	(0.22, 3.80)	1.32	(0.69, 2.52)
Managing finances	1.25	(0.84, 1.87)	1.16	(0.78, 1.73)	0.92	(0.31, 2.73)
Managing health care	0.85	(0.50, 1.45)	0.83	(0.50, 1.38)	0.84	(0.51, 1.39)
Phone use	0.90	(0.60, 1.35)	0.79	(0.54, 1.16)	0.82	(0.56, 1.19)
Shopping	1.31	(0.72, 2.38)	1.02	(0.64, 1.64)	0.93	(0.61, 1.43)
Travel	0.92	(0.06, 14.99)	**0.47**	**(0.16, 1.39)**	0.69	(0.33, 1.45)
**Speed vs. Control**						
Preparing meals	1.33	(0.31, 5.72)	1.01	(0.36, 2.86)	1.41	(0.68, 2.94)
Housework	0.97	(0.55, 1.71)	2.53	(0.79, 8.15)	1.04	(0.54, 2.02)
Managing finances	0.85	(0.57, 1.28)	0.87	(0.58, 1.31)	1.01	(0.36, 2.84)
Managing health care	1.25	(0.76, 2.05)	1.16	(0.72, 1.85)	1.20	(0.75, 1.91)
Phone use	0.90	(0.61, 1.33)	0.84	(0.58, 1.22)	0.80	(0.55, 1.16)
Shopping	1.39	(0.78, 2.49)	1.15	(0.73, 1.81)	0.98	(0.65, 1.48)
Travel	4.94	(0.58, 41.86)	0.88	(0.36, 2.17)	1.22	(0.64, 2.29)

*IADL, Instrumental Activities of Daily Living; OR, odds ratio; CI, confidence interval*.

*The OR of the intervention group being more likely to experience first IADL difficulty in each task group compared to the control group; statistically significant results are displayed in bold. OR <1 indicates that individuals in the intervention group were less likely to experience first IADL difficulty in a specific task group compared to the control group. OR > 1 indicates that individuals in the intervention group were more likely to experience first IADL difficulty in a specific task group compared to the control group*.

## Discussion

### Main Findings

In this study, we empirically tested the relative ordering of incident difficulty of seven groupings of IADLs (preparing meals, housework, managing finances, managing health care, phone use, shopping, and travel outside of the home) in a large sample of high-functioning community-living older adults. Overall, the probability of incident IADL difficulty at any one specific age between 65 and 80 years is low, but increases steadily with age, particularly for housework, managing health care, and phone use. Managing health care and phone use represent cognitively demanding tasks, while housework represents more physically demanding tasks.

Population-based cohort studies (14, 15) have reported that difficulty in shopping and housework became difficult first in older adults. While we also found housework to be the most likely to become difficult first in our cohort, the hazard of incident difficulty in both shopping and traveling outside the home was low. This difference in findings may be attributable to selection effects into the ACTIVE study sample. Most participants had to travel to a research facility for assessments and training, necessitating the ability to travel outside the home. Ability to travel is also an essential task for shopping. Despite this, if travel is indeed relatively preserved in the community-living populations of older adults, this has implications for the feasibility of community-based cognitive training interventions, perhaps those with social components, as opposed to self-guided interventions at home.

The steady increase in the probability of incident difficulty with health care tasks is important, given that older adults with multimorbidities require complex health care management (33). Difficulty managing medications is associated with increased hospital admissions, health care costs, and mortality (34). Older adults with memory loss may rate themselves as compliant with medications even when scoring worse on a medication management test compared to healthy peers (35), indicating the early need for assistance with medications. In our study, self-reported incident difficulty with medication management was low but may have been underreported for this reason. Moreover, our study only assessed self-care, but complex health care policies are similarly challenging to navigate for older adults with cognitive impairment (36). Access to care in combination with assistive devices and environmental adaptations could all affect an individual's self-reported difficulty.

It is important to consider that many IADL tasks are sex stereotyped. For example, women traditionally perform cooking and cleaning-related tasks, while men traditionally handle finances. The original developers of early IADL scales suggested using differential scoring for males and females for this reason (6). Trends in IADL difficulty and performance across birth cohorts have shown that the younger cohorts of older adults are less likely to exhibit stereotyped behaviors (37). It is possible that because the majority of subjects in this study are female, we observed little increase in incident difficulty of managing finances and a steady increase in difficulty with housework, although this does not explain why little increase in difficulty with preparing meals was observed.

### Cognitive Intervention Effect

Patterns in IADL difficulty may be associated with trajectories of cognitive decline. It is unclear whether cognitive decline or IADL impairment occurs first. Evidence from the ACTIVE study has suggested that deterioration in self-reported difficulty in IADLs precede decline in tests of memory, reasoning, and speed of processing (38); other studies have shown that cognitive impairment predicts future IADL difficulty (39). Executive functioning, in particular, has emerged as an important contributor in ability to perform IADLs (26, 40–45). There was no difference in the first IADL difficulty in any of the cognitive training groups relative to the control group in our study. We found no evidence from our analysis to recommend a specific cognitive intervention type to delay IADL difficulty onset; rather, identification of incident cognitively demanding IADL impairment may help select individuals most likely to benefit from cognitive intervention. We plan to further investigate the relationship between incident IADL difficulty and cognitive decline across cognitive intervention groups in future studies.

Although IADL performance is typically worse in persons with mild cognitive impairment (MCI) compared to healthy older adults, it is unclear which IADLs decline first in MCI (46), potentially because specific IADL difficulties may be related to MCI subtype (47). Generally, difficulty with finances has been associated with future progression to dementia, and difficulty with medication use and household activities has been associated with prospective and temporal order memory (48).

Our findings do not contradict previous studies within the ACTIVE cohort showing that cognitive training improves overall self-reported IADL difficulty (26, 49). Previous analyses included individuals with prevalent IADL difficulty at baseline, rather than limiting to individuals who entered the study free of any difficulty. Our study only included individuals with no reported IADL difficulty at baseline, and so, they could not improve on the IADL difficulty scale.

### Limitations

One limitation of this study is the use of self-reported, rather than objective or informant-reported, measures of IADL difficulty. Self-reported limitations in older adults do not always match actual performance of the same task (50). However, performance-based measures were collected for several IADLs and may be used in future analyses to confirm results. Another important limitation is that it is not known in this study whether adults were using assistive devices that could impact reported task difficulty. For example, keeping track of doctor appointments was rarely the first incident difficult task, perhaps due to use of calendars.

One challenge in this study was the discrete nature of the data collection; follow-up of participants only occurred at years 1, 2, 3, 5, and 10. Although the majority of participants had only one first incident IADL difficulty, a substantial number of people experienced more than one first incident IADL at a study visit. Likely, these IADL difficulties occurred in some order, but this order was unobserved due to it occurring between visits. Changing the analysis time-scale to age and using discrete time follow-up methods were used to combat this challenge.

Because of the large number of IADL tasks (19) collected in this study, related tasks were combined *a priori* into seven task groups in order to facilitate model fitting and parsimony of result interpretation. However, tasks within a task group are diverse and involve a variety of different skills. It is possible that some of the variation in skills represented is lost when collapsing the 19 tasks into seven task groups.

Importantly, this study considers first incident IADLs across an entire sample of older adults. It is likely that the first incident IADL varies based on whether an individual is experiencing cognitive deterioration, physical deterioration, or both. Future studies should address this heterogeneity by identifying patterns of first incident IADL difficulty across subgroups of individuals.

## Conclusion

The IADLs with the greatest probability of incident difficulty in this study were housework, managing health care, and phone use, and the probability of incident difficulty increased with age. Although patterns of early difficulty were heterogenous in this study and in others, early identification of task difficulty allows for intervention that can reduce health care expenditure and poor outcomes.

## Data Availability Statement

Publicly available datasets were analyzed in this study. This data can be found here: https://www.icpsr.umich.edu/icpsrweb/ICPSR/studies/4248/datadocumentation.

## Ethics Statement

Ethical review and approval was not required for the study on human participants in accordance with the local legislation and institutional requirements. The patients/participants provided their written informed consent to participate in this study.

## Author Contributions

DF and AG were involved in the study design and data analysis. DF drafted the initial manuscript. All authors provided critical manuscript reviews and edits.

## Conflict of Interest

The authors declare that the research was conducted in the absence of any commercial or financial relationships that could be construed as a potential conflict of interest.
