# Supplementary material for: Incident Instrumental Activities of Daily Living Difficulty in Older Adults: Which Comes First? Findings From the Advanced Cognitive Training for Independent and Vital Elderly Study
Source: Front Neurol. 2020 Oct 22;11:550577. doi: 10.3389/fneur.2020.550577 (PMC7642324; doi:10.3389/fneur.2020.550577)
Supplement: Supplementary file 1 [file Data_Sheet_1.DOCX]

Supplemental Tables and Figures

Supplemental Figure 1. Hazard of Incident IADL Difficulty by Task Group, Unadjusted Model: Results from ACTIVE (N=1,277)


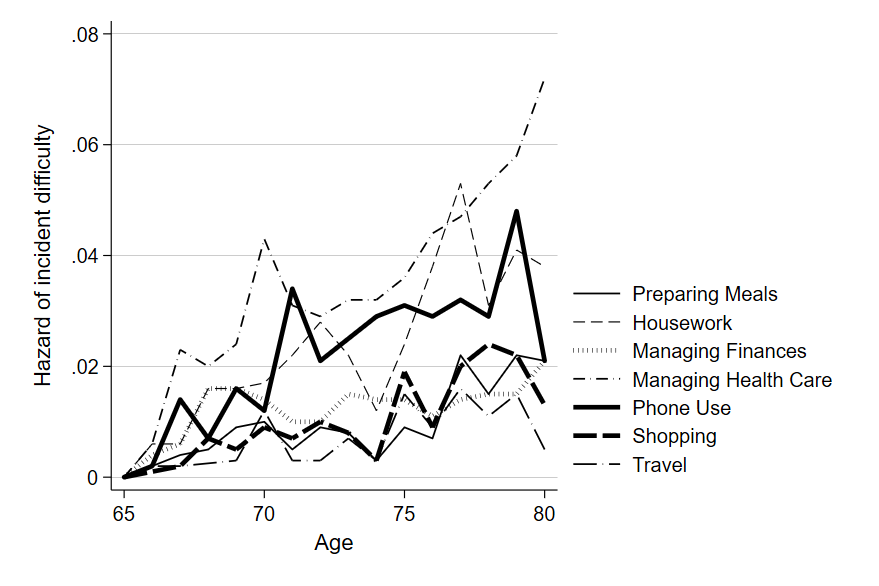


Legend. Results of MEPSUM model, as described in the Methods section. Figure shows the hazard estimates of incident difficulty in seven Instrumental Activities of Daily Living (IADL) task groups as a function of age. The hazards are estimated from a model with age 65 years as the time origin.

Supplemental Figure 2. Probability of Remaining Free of IADL Difficulty by Task Group, Unadjusted Model: Results from ACTIVE (N=1,277)


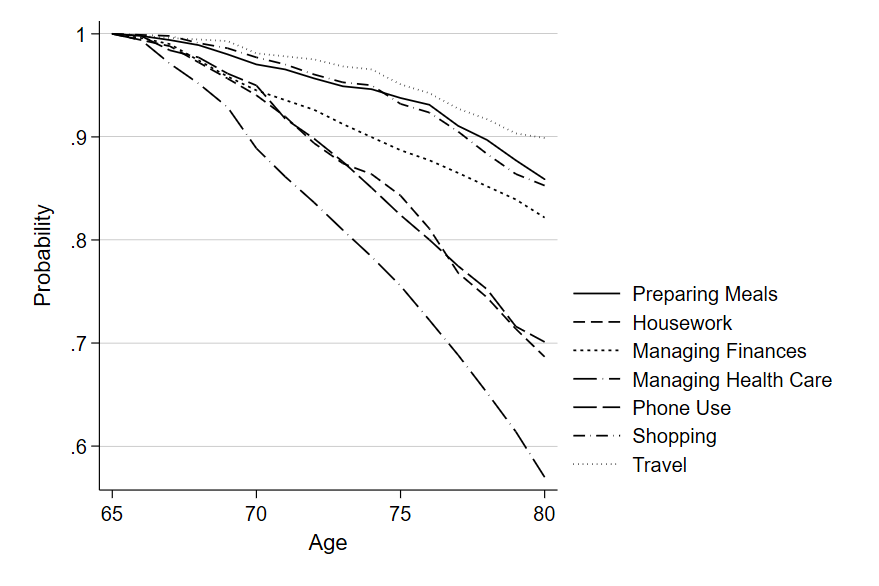


Legend. Results of MEPSUM model, as described in the Methods section. Figure shows the probability of remaining free of difficulty in seven Instrumental Activities of Daily Living (IADL) task groups as a function of age. This survival probability is derived using model hazard estimates in the standard discrete time survival formula (see ref. 28).
